# Supplementary material for: Ultrahigh loading dry-process for solvent-free lithium-ion battery electrode fabrication
Source: Nat Commun. 2023 Mar 10;14:1316. doi: 10.1038/s41467-023-37009-7 (PMC10006413; doi:10.1038/s41467-023-37009-7)
Supplement: Supplementary file 1 — Supplementary Information [file 41467_2023_37009_MOESM1_ESM.pdf]

Supplementary Information

Ultrahigh loading dry-process for solvent-free lithium-ion battery electrode fabrication

*Minje Ryu, Young-Kuk Hong, Sang-Young Lee and Jong Hyeok Park**

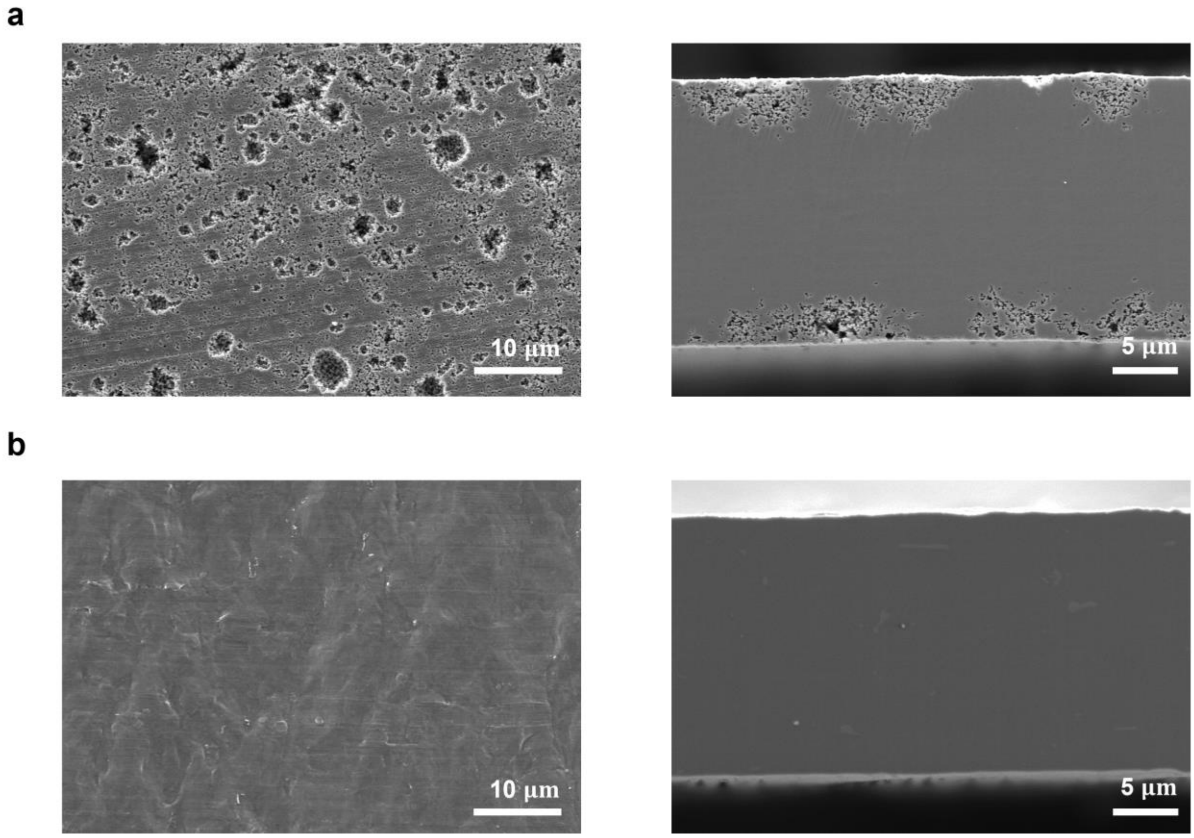

Supplementary Figure 1 | Comparison of the current collectors. Top and cross-section SEM images of **a** etched Al foil, and **b** normal Al foil.

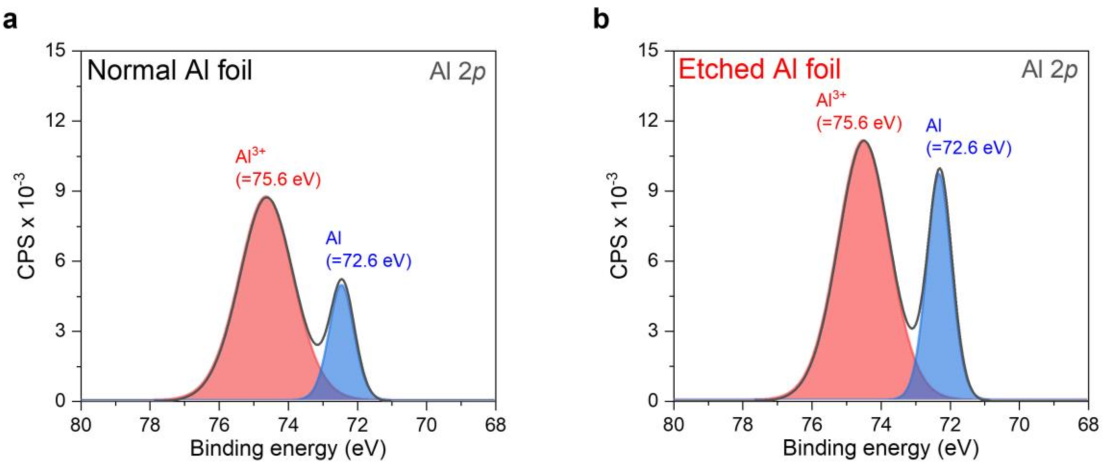

Supplementary Figure 2 | XPS Al 2*p* spectra of **a** the normal Al foil and **b** the etched Al foil (fitted with 30% Gaussian/Lorentzian algorithm).

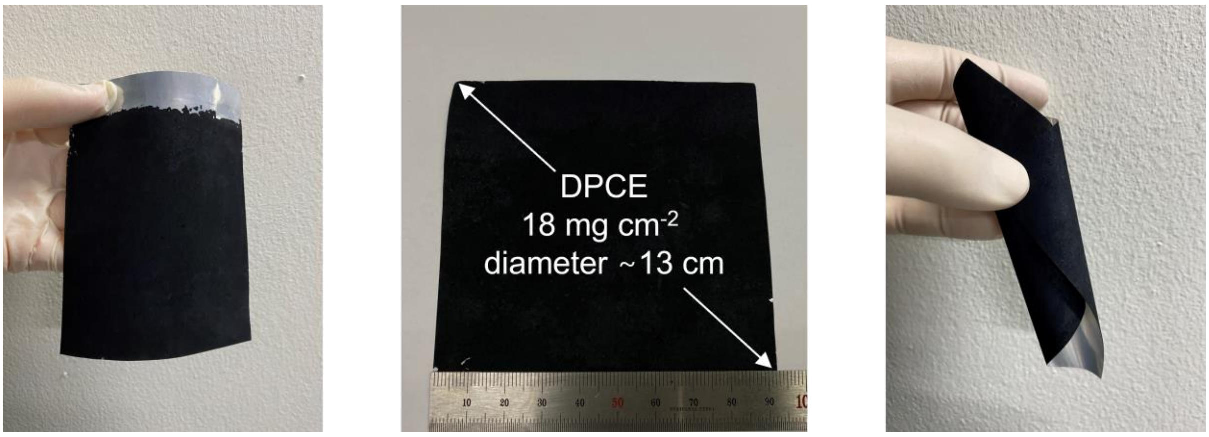

Supplementary Figure 3 | Digital image of the as-prepared large-area DPCE.

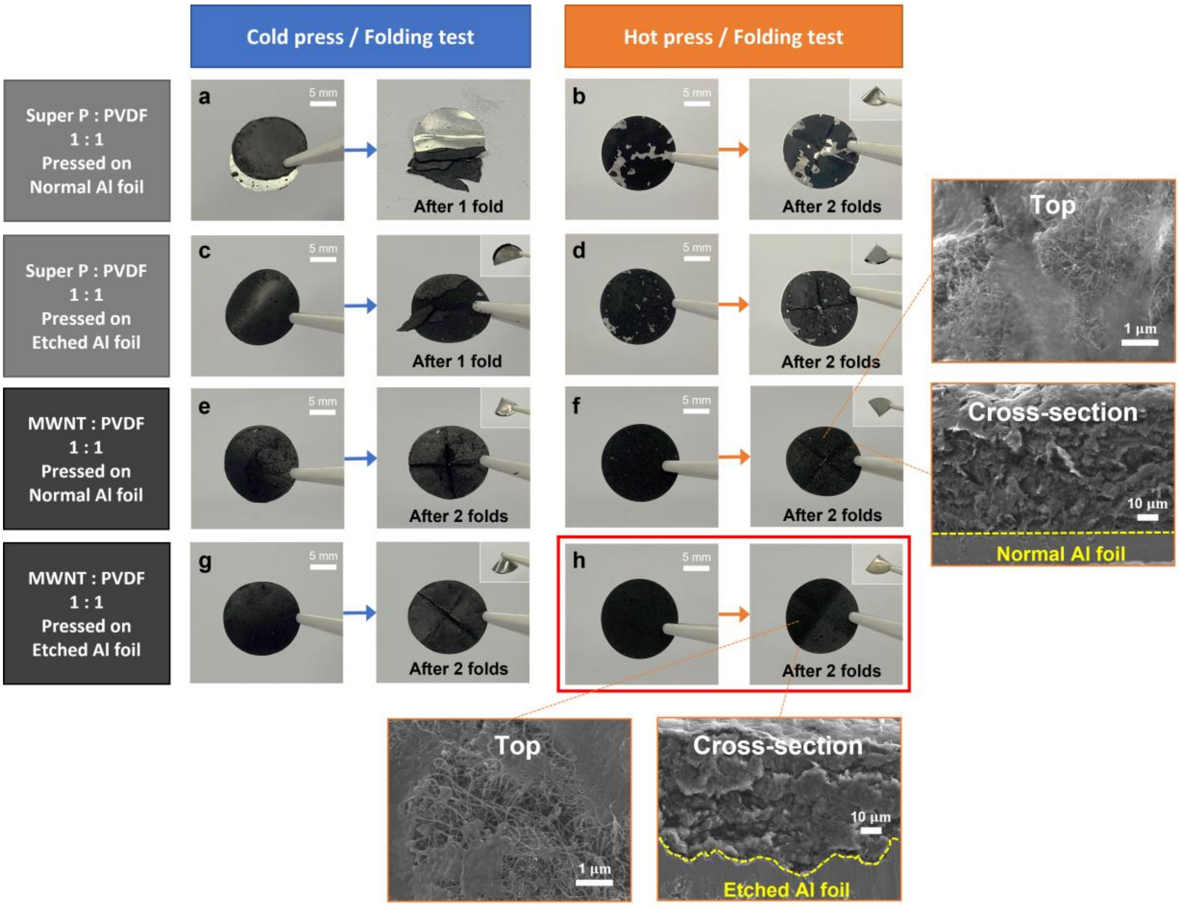

Supplementary Figure 4 | Comparison of the dry press-coating capability of the conductive scaffolds. **a**–**d** Folding test of Super P:PVDF–1:1 electrode after cold/hot press on normal Al foil and etched Al foil. **e**–**h** Folding test of MWNT:PVDF–1:1 electrode after cold/hot press on normal and etched Al foil, the diameter of the disc is 15 mm.

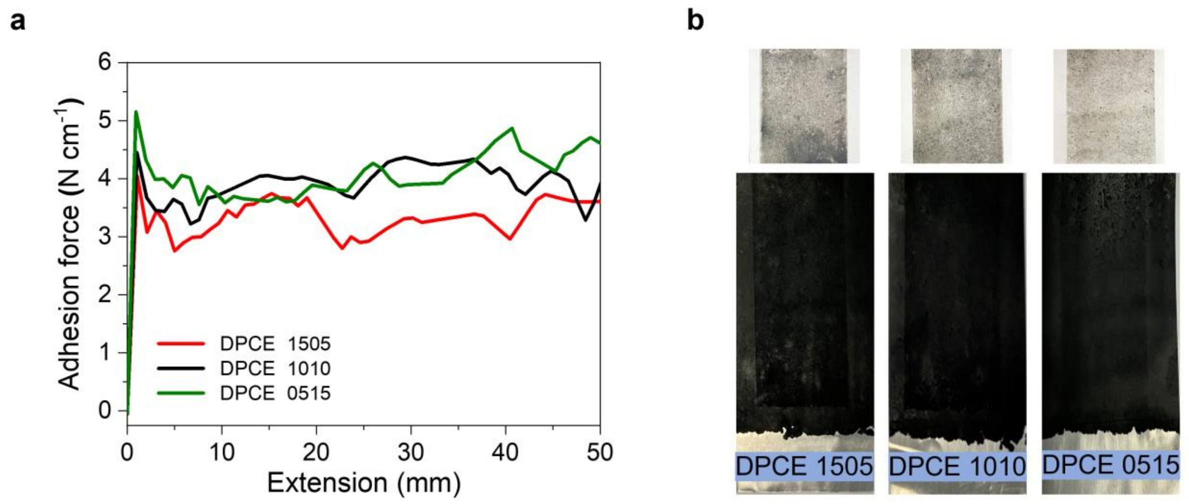

Supplementary Figure 5 | The mechanical properties of the DPCE. **a** Peeling force and extension curves. **b** Images of the stripped electrodes after the peeling test.

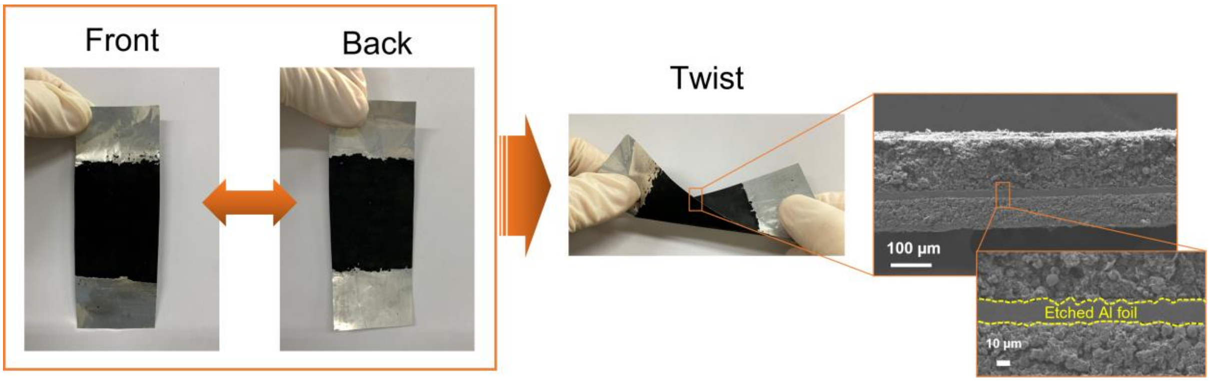

Supplementary Figure 6 | The mechanical properties of the DPCE. Dry press-coating on both sides of the etched Al foil and the cross-section SEM image of the double-sided DPCE after twisting.

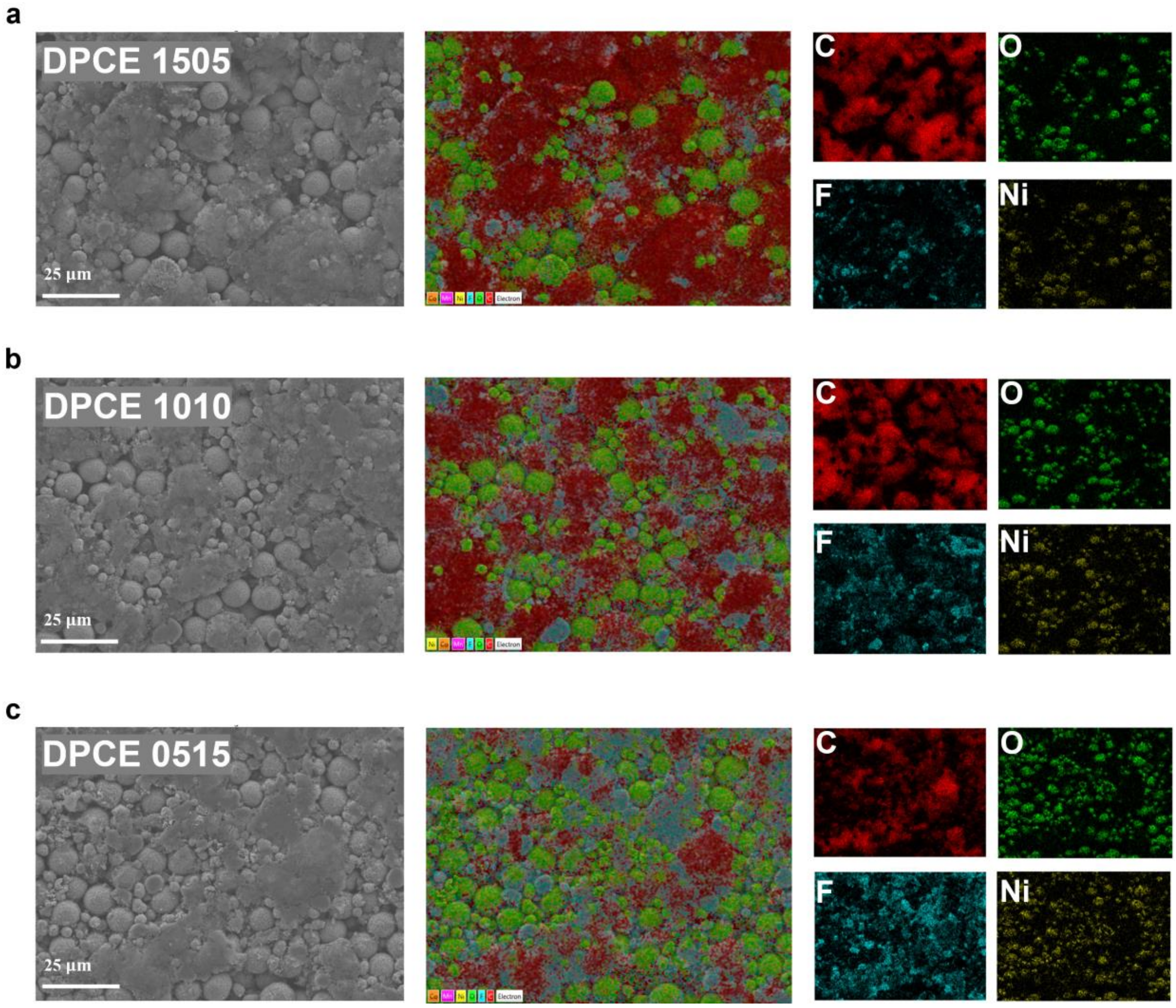

Supplementary Figure 7 | Top SEM and the elemental mapping images of the DPCE with varying MWNT and binder content: **a** DPCE 1505, **b** DPCE 1010 and **c** DPCE 0515.

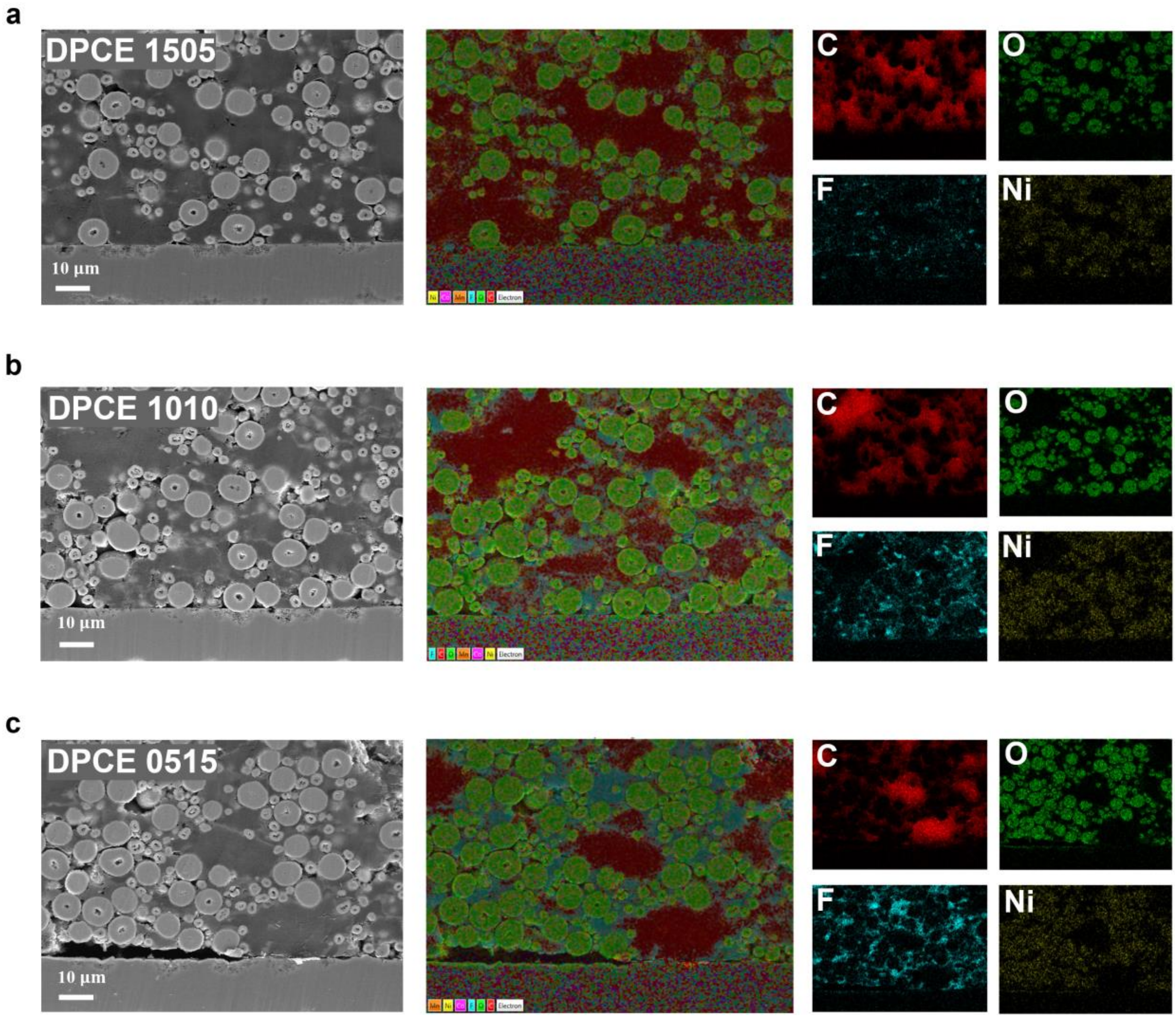

Supplementary Figure 8 | Cross-section SEM and the elemental mapping images of the DPCE with varying MWNT and binder content: **a** DPCE 1505, **b** DPCE 1010 and **c** DPCE 0515.

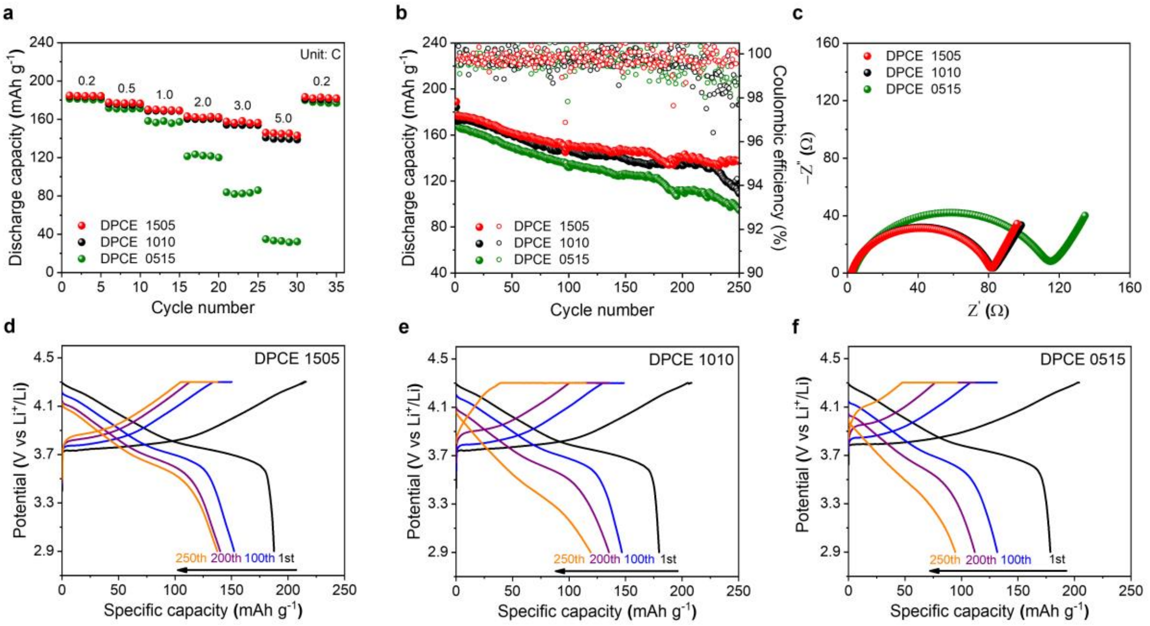

Supplementary Figure 9 | The electrochemical performance of the DPCE with varying MWNT and PVDF content. **a** Discharge rate capability over a range of different current densities (0.2–5.0 C). **b** Cycle performance and corresponding coulombic efficiency at 0.5 C. **c** Nyquist plots of pristine DPCEs. **d**-**f** Charge/discharge voltage profile of DPCE 1505 (**d**), DPCE 1010 (**e**) and DPCE 0515 (**f**).

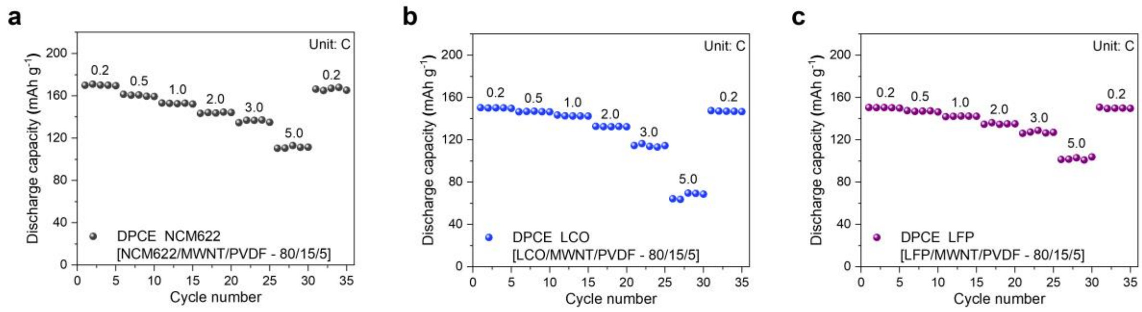

Supplementary Figure 10 | C-rate performances of the DPCE with different active materials: **a** NCM622, **b** LCO, and **c** LFP.

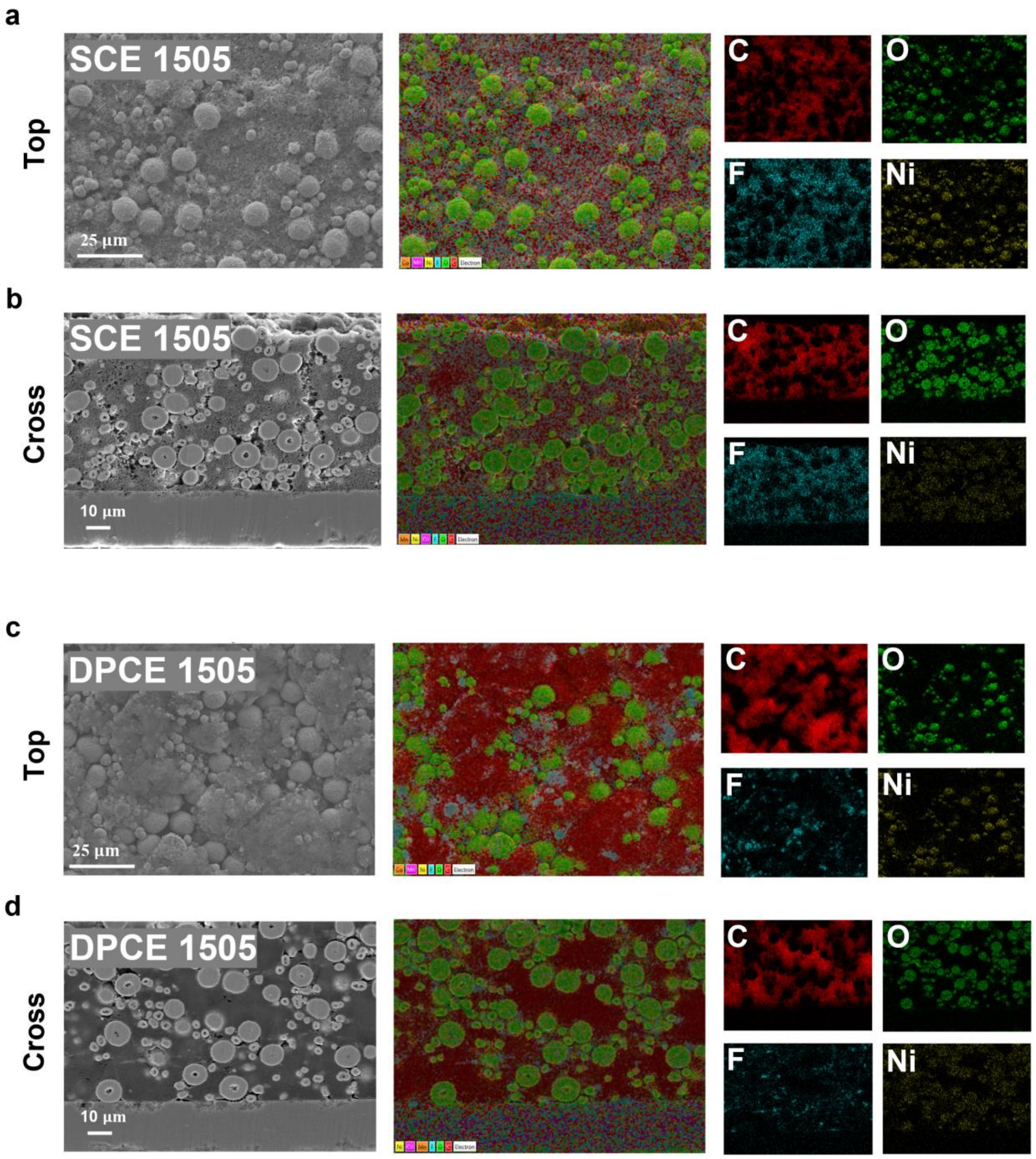

Supplementary Figure 11 | Comparison of the SEM and elemental mapping images of the SCE: **a** top, and **b** cross-section and DPCE: **c** top, and **d** cross-section.

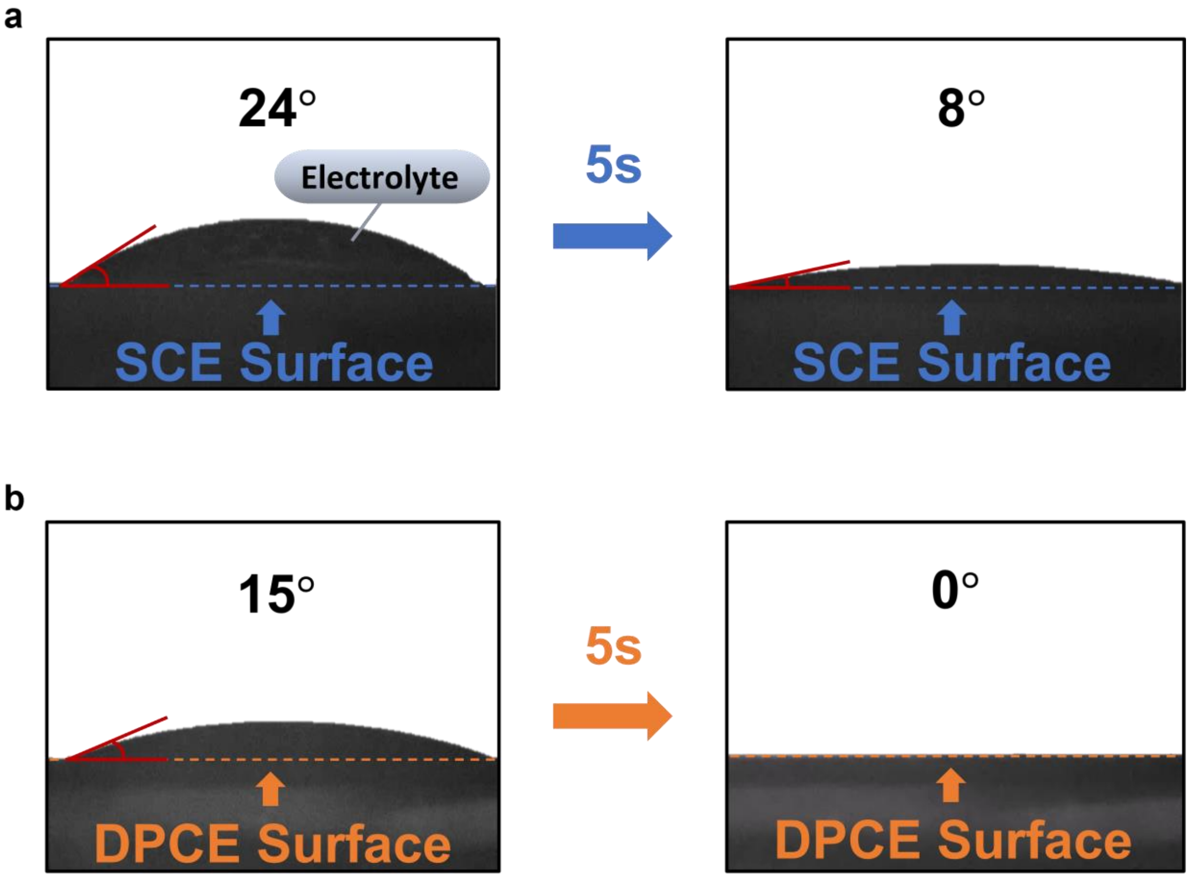

Supplementary Figure 12 | Comparison of the contact angle of **a** the SCE and **b** DPCE before and after 5s.

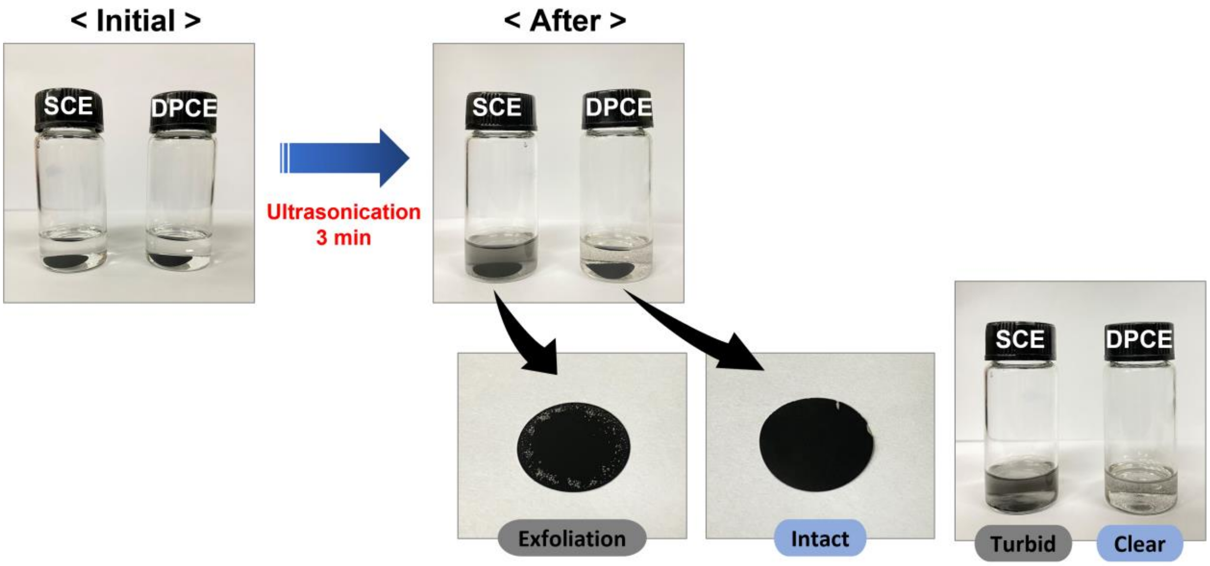

Supplementary Figure 13 | Comparison of the electrolyte swelling of the DPCE and SCE.

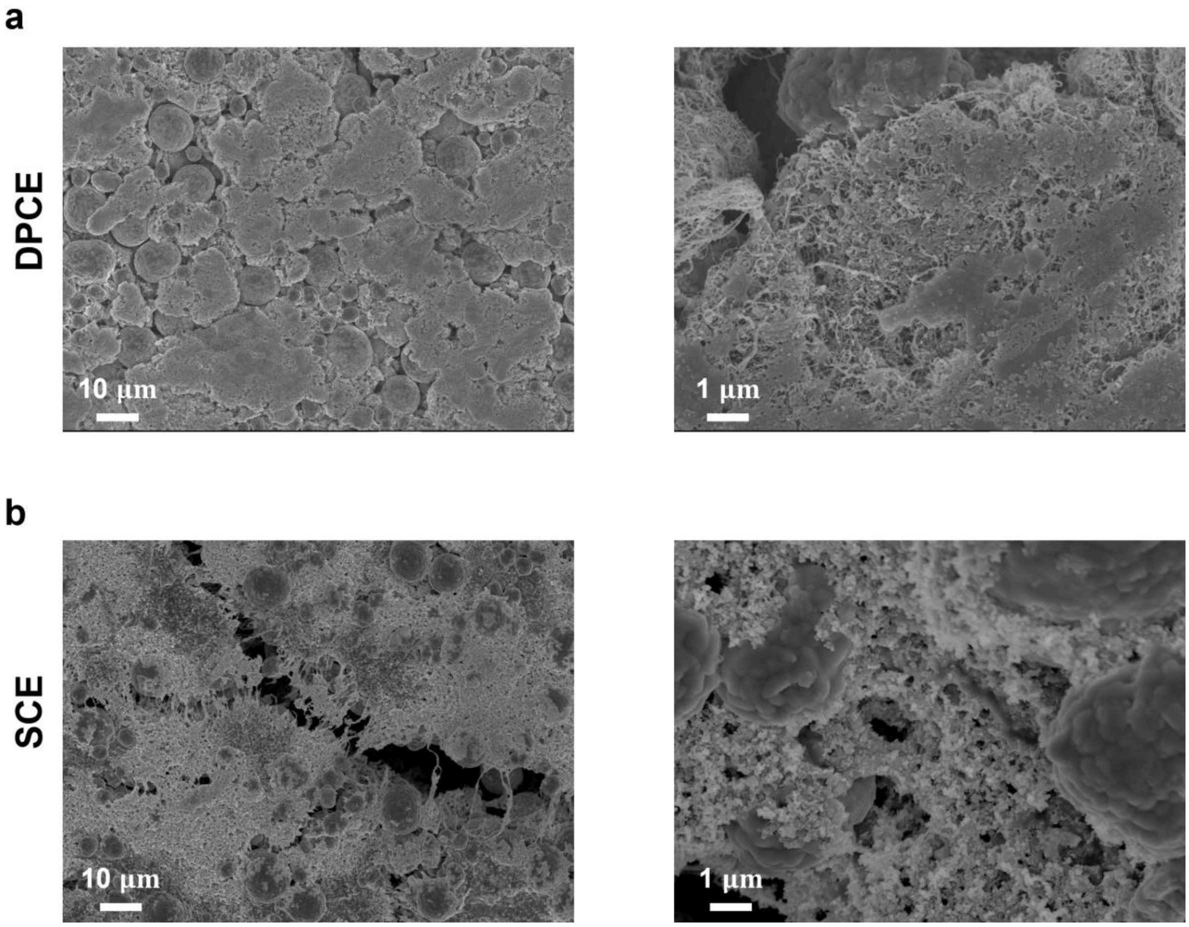

Supplementary Figure 14 | Comparison of the top SEM images of **a** the DPCE, and **b** SCE after 100 cycles under different magnification.

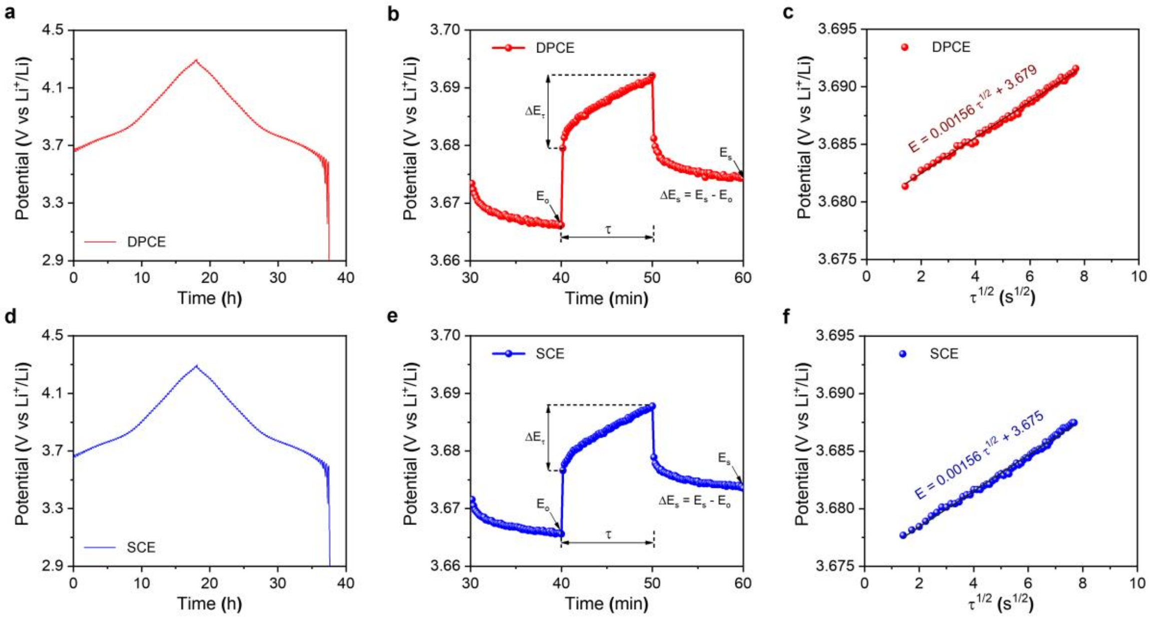

Supplementary Figure 15 | GITT experiment for the DPCE and SCE. **a**, **d** GITT profile of DPCE (18 mg cm^{-2}), and SCE (13 mg cm^{-2}) for the second cycle (at a current density of 0.1 C, with the interruption time between the pulses is 10 min). **b**, **e** GITT profile showing the 3rd charging step of DPCE and SCE. **c**, **f** Linear regression line showing the linear relationship between the cell potential and $\tau^{1/2}$ for DPCE and SCE.

The diffusion coefficients of the DPCE and SCE are calculated based on the following equation:

$$D = \frac{4}{\pi\Delta\tau}\left(\frac{m_B V_M}{M_B S}\right)^2\left(\frac{\Delta E_S}{\Delta E_\tau}\right)^2 \qquad (1)$$

where S is the area of the electrode; m_B, M_B, V_M are the mass, molar mass, and molar volume of the active material (NCM712), respectively. In this work, the M_B = 96.91 g mol^{-1}, V_M = 49.37 cm^3 mol^{-1} (DPCE) / 39.06 cm^3 mol^{-1} (SCE), and m_B/S is the areal mass loading of NCM712. Other parameters in the equation are displayed in the figure.

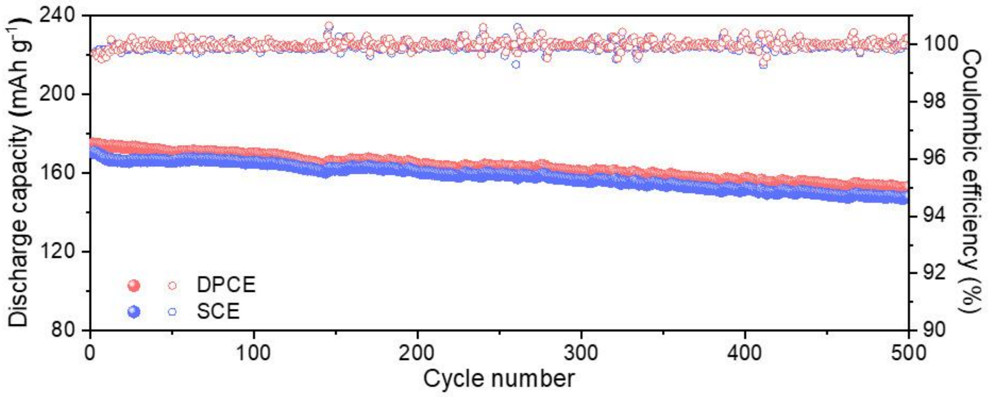

Supplementary Figure 16 | Cycling performance of the DPCE and SCE full cell (graphite electrode as a counter electrode) at 1.0 C with corresponding coulombic efficiency.

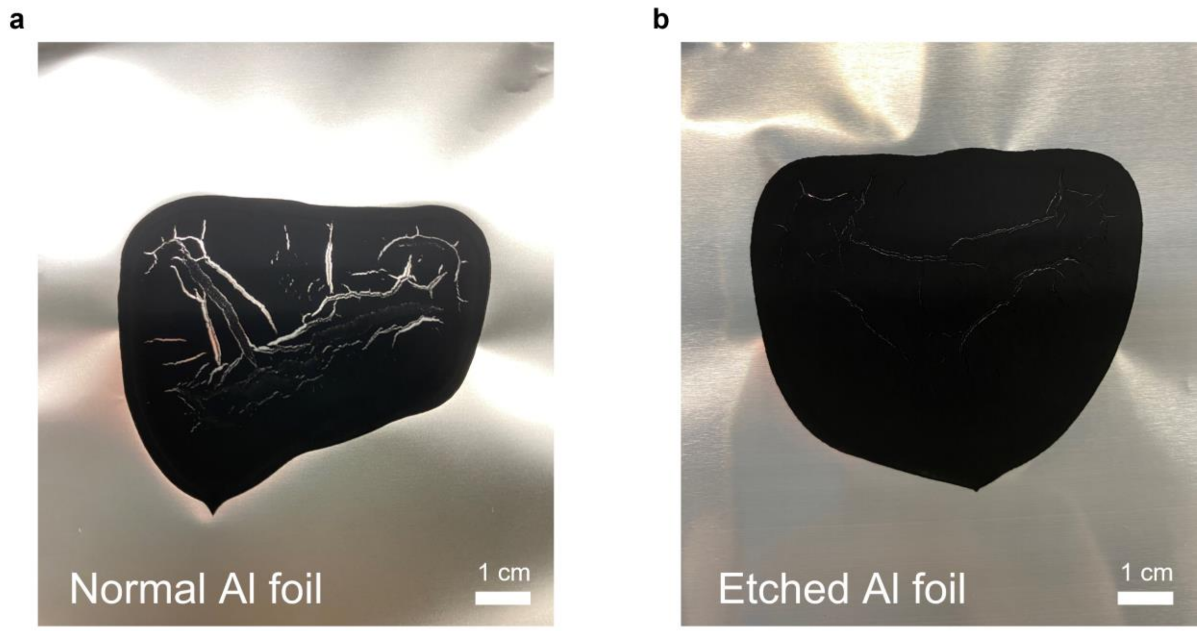

Supplementary Figure 17 | Morphological comparison of the high-loading slurry-coated electrode (HL-SCE) ($\geq$ 16 mg cm^{-2} (corresponding to ~ 80 μm)) on **a** normal Al foil vs. **b** etched Al foil. (NCM712/Super P/PVDF – 80/15/5 (w/w/w))

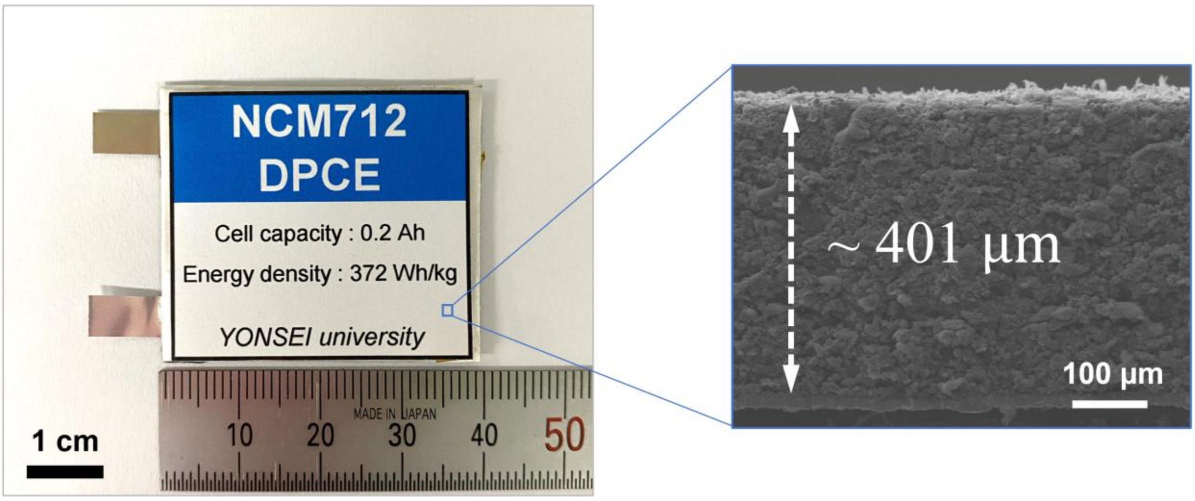

Supplementary Figure 18 | Digital image of the as-prepared Li-metal pouch cell (40 × 35 mm^2 in size) installed with DPCE (areal mass loading/areal capacity/thickness of 70 mg cm^{-2}/13.2 mAh cm^{-2}/401 μm) coupled with Li-metal anode (200 μm) (cell capacity and specific energy was calculated without including the cell package weight).

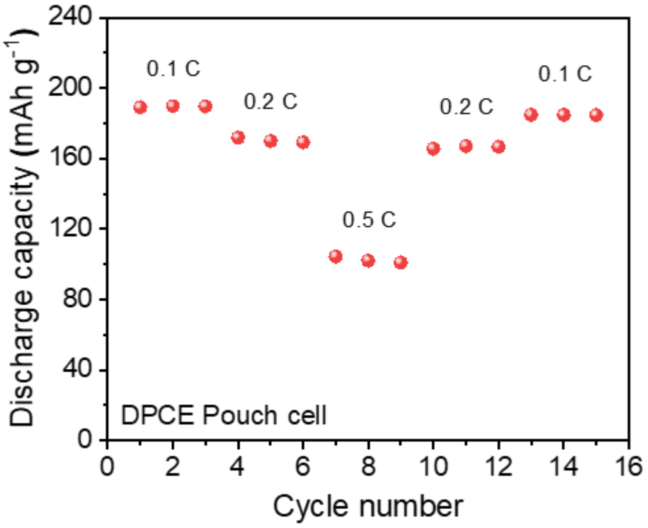

Supplementary Figure 19 | C-rate performance of the DPCE Li metal pouch cell (areal mass loading of 70 mg cm^{-2}, corresponding to 13.2 mAh cm^{-2}) under different current densities (0.1 C (= 1.35 mA cm^{-2}) – 0.5 C (= 6.76 mA cm^{-2})) at a voltage range of 2.9–4.3 V.

Supplementary Table 1 | Calculation details for the specific energies and volumetric energy densities of the Li metal cells equipped with the DPCEs.

The specific energy of the Li metal cell was calculated based on the following equation:

$$\text{Specific energy (Wh kg}^{-1}) = \frac{\text{Energy}}{\text{Mass of cell}} = \frac{\frac{\text{Energy}}{\text{Area}}}{\frac{\text{Mass of cell}}{\text{Area}}}$$

$$= \frac{\text{Nominal voltage} \times \text{C/A}}{\text{M}_{\text{cathode}}/\text{A} + \text{M}_{\text{anode}}/\text{A} + \text{M}_{\text{separator}}/\text{A} + \text{M}_{\text{electrolyte}}/\text{A} + \text{M}_{\text{package}}/\text{A}}$$

where $M_{cathode}$, M_{anode}, $M_{separator}$, $M_{electrolyte}$ and $M_{package}$ denote the mass of cathode (including the etched Al current collector (20 μm)), mass of anode (comprised of Li metal (200 μm) and Cu current collector (18 μm)), mass of PE separator, mass of injected electrolyte, and mass of pouch-cell package, respectively (C and A refer to the capacity and area, respectively).

C/A [mAh cm^{-2}]	Nominal voltage [V]	$M_{cathode}$/A [mg cm^{-2}]	M_{anode}/A [mg cm^{-2}]	$M_{separator}$/A [mg cm^{-2}]	$M_{electrolyte}$/A [mg cm^{-2}]	$M_{package}$/A [mg cm^{-2}]	M_{total}/A [mg cm^{-2}]	Specific energy [Wh kg^{-1}]
17.6	3.9	105.4	26.8	0.8	36.0	21.3	190.3	360

The volumetric energy density of the Li metal cell was calculated based on the following equation:

$$\text{Volumetric energy density (Wh L}^{-1}) = \frac{\text{Energy}}{\text{Thickness of cell}} = \frac{\text{Nominal voltage} \times \text{C/A}}{\text{T}_{\text{cathode}} + \text{T}_{\text{anode}} + \text{T}_{\text{separator}} + \text{T}_{\text{package}}}$$

where $T_{cathode}$, T_{anode}, $T_{separator}$ and $T_{package}$ are the thickness of cathode, thickness of anode, thickness of PE separator, and thickness of pouch-cell package, respectively (measured using a micrometer).

C/A [mAh cm^{-2}]	Nominal voltage [V]	$T_{cathode}$ [μm]	T_{anode} [μm]	$T_{separator}$ [μm]	$T_{package}$ [μm]	T_{total} [μm]	Volumetric energy density [Wh L^{-1}]
17.6	3.9	593	218	18	150	979	701

Supplementary Table 2 | Comparison of the electrochemical performances between the DPCE (this work) and previously reported solvent-free dry electrodes

**Methods**	**Active materials**	**Composition ratio [active materials: conductive agent:binder]**	**Thickness [μm]**	**Mass loading [mg cm^{-2}]**	**Areal capacity [mAh cm^{-2}]**	**Ref.**
Dry press-coating	NCM712	80:15:5	573	100	17.6 (0.1 C)	This work
Dry press (Cold)	NCM523	1:9:0	-	26	3.2 (0.1 C)	S1
Dry press (Cold)	LFP	1:1:0	340	11	1.9 (0.1 C)	S2
Electrostatic dry powder coating	NCM111	19:1:1	40	10	1.5 (0.2 C)	S3
Electrostatic spray deposition + Hot roll press	NCM111	90:5:5	60	-	-	S4
	LCO	98:1:1	-	-	-	S5
Electrostatic spraying	NCM523	87.5:7.5:5	12	3	0.5 (1.0 C)	S6
Continuous molding method + Hot roll press	NCM (Not specified)	90:5:5	200	65	9.1 (0.1 C)	S7

Supplementary Table 3 | Comparison of the electrochemical performances between the DPCE (this work) and previously reported high mass loading electrodes

Methods	Active materials	Composition ratio [active materials: conductive agent:binder]	Thickness [µm]	Mass loading [mg cm^{-2}]	Areal capacity [mAh cm^{-2}]	Specific energy [Wh kg^{-1}]	Energy density [Wh L^{-1}]	Ref.
Dry press-coating	NCM712	80:15:5	573	100	17.6 (0.1 C)	360	701	This work
Slurry coating	LFP	8:1:1	320	29	4.6 (0.1 C)	-	-	S8
	NCM811	97:2:1	95	27	5.2 (0.2 C)	-	-	S9
	NCM111	90:7:3	305	82	9.2 (0.1 C)	188	436	S10
	NCM622	92:4:4	154	38	6.1 (0.1 C)	246	-	S11
Directional freezing	LFP	5:1:0	2000	34	5.1 (0.2 C)	235	-	S12
3D foam	NCM (Not specified)	84:7:9	1200	89	10.0 (0.05 C)	210	-	S13
Slurry infiltration into Al-foam	NCM111	97:2:1	260	27	7.0 (0.1 C)	350	700	S14
Electrospinning + Airbrush spraying	LFP	9:1:0	577	79	12.2 (0.02 C)	-	-	S15

Supplementary references

1. Walker, B. A. et al. Dry-pressed lithium nickel cobalt manganese oxide (NCM) cathodes enabled by holey graphene host. *Electrochim. Acta* **362,** 137129 (2020).
2. Kirsch, D. J. et al. Scalable Dry Processing of Binder-Free Lithium-Ion Battery Electrodes Enabled by Holey Graphene. *ACS Appl. Energy Mater.* **2**, 2990–2997 (2019).
3. Al-Shroofy, M. et al. Solvent-free dry powder coating process for low-cost manufacturing of LiNi1/3Mn1/3Co1/3O2 cathodes in lithium-ion batteries. *J. Power Sources* **352**, 187–193 (2017).
4. Ludwig, B., Zheng, Z., Shou, W., Wang, Y. & Pan, H. Solvent-Free Manufacturing of Electrodes for Lithium-ion Batteries. *Sci. Rep.* **6**, 23150 (2016).
5. Ludwig, B. et al. Understanding Interfacial-Energy-Driven Dry Powder Mixing for Solvent-Free Additive Manufacturing of Li-Ion Battery Electrodes. *Adv. Mate. Interfaces* **4**, 1700570 (2017).
6. Zhen, E. et al. Effects of binder content on low-cost solvent-free electrodes made by dry-spraying manufacturing for lithium-ion batteries. *J. Power Sources* **515**, 230644 (2021).
7. Liu, J. et al. Scalable Dry Printing Manufacturing to Enable Long-Life and High Energy Lithium-Ion Batteries. *Adv. Mater. Technol.* **2**, 1700106 (2017).
8. Zhang, Y. et al. Polymer Molecular Engineering Enables Rapid Electron/Ion Transport in Ultra-Thick Electrode for High-Energy-Density Flexible Lithium-Ion Battery. *Adv. Funct. Mater.* **31**, 2100434 (2021).
9. Kim, N. Y. et al. Amphiphilic Bottlebrush Polymeric Binders for High-Mass-Loading Cathodes in Lithium-Ion Batteries. *Adv. Energy Mater.* **12**, 2102109 (2022).
10. Singh, M., Kaiser, J. & Hahn, H. A systematic study of thick electrodes for high energy lithium ion batteries. *J. Electroanal. Chem.* **782**, 245–249 (2016).
11. Gallagher, K. G. et al. Optimizing Areal Capacities through Understanding the Limitations of Lithium-Ion Electrodes. *J. Electrochem. Soc.* **163**, A138–A149 (2016).
12. Kang, S. et al. Stretchable Lithium-Ion Battery Based on Re-entrant Micro-honeycomb Electrodes and Cross-Linked Gel Electrolyte. *ACS Appl. Mater. Interfaces* **14**, 3660-3668 (2020)

13. Wang, J. S., Liu, P., Sherman, E., Verbrugge, M. & Tataria, H. Formulation and characterization of ultra-thick electrodes for high energy lithium-ion batteries employing tailored metal foams. *J. Power Sources* **196**, 8714–8718 (2011).
14. Fritsch, M., Standke, G., Heubner, C., Langklotz, U. & Michaelis, A. 3D-cathode design with foam-like aluminum current collector for high energy density lithium-ion batteries. *J. Energy Storage* **16**, 125–132 (2018).
15. Huang, H. et al. General Airbrush-Spraying/Electrospinning Strategy for Ultrahigh Areal-Capacity LiFePO4-Based Cathodes. *ChemElectroChem* **5**, 2330–2335 (2018).
